# USABILITY OF AN AFFECT-AWARE MULTIMODAL PERSONALIZED INTERACTIVE MODEL AMONG FAMILY CAREGIVERS

**DOI:** 10.1093/geroni/igae098.2909

**Published:** 2024-12-31

**Authors:** Yu-Ping Chang, Aditya Kumar Dwibedi, Bhushan Mahajan, Skylar Bard, Sreyasee Das Bhattacharjee

**Affiliations:** University at Buffalo, SUNY, Buffalo, New York, United States; University at Buffalo, SUNY, Buffalo, New York, United States; University at Buffalo, SUNY, Buffalo, New York, United States; University at Buffalo, SUNY, Buffalo, New York, United States; University at Buffalo, SUNY, Buffalo, New York, United States

## Abstract

Long-term family caregiving for people with dementia is often exhausting and poses risks to caregivers’ overall health. Most informal caregivers are not well-equipped to perform their caregiving responsibilities at home, facing numerous challenges, feeling stressed and overwhelmed, and receiving limited support. Artificial Intelligence, specifically following the release of ‘generative pre-trained transformer’ (GPT), has demonstrated promise in delivering assistive technology solutions in various domains in healthcare. Our project aims to evaluate the usability of an affect-aware multimodal interactive prototype that can facilitate human-like sensemaking, providing emotional support, and generating personalized recommendations to family caregivers. The prototype can receive multimodal inputs (speech, text) from family caregivers and evaluate their personalized emotional and task-related contexts to deliver short, machine-driven, affect-aware messages augmented with multimodal (text, video) recommendations on a pre-defined set of topics. Six healthcare providers and family caregivers were invited to evaluate the prototype. They were instructed to test the prototype on a computer for 1.5 to 2 hours and provide feedback regarding their experiences (e.g., content, accuracy, format, and technology). Participants commented on the usefulness and accuracy of information generated by the model for supporting family caregivers, the level of personalization, the level of detail for various caregiving tasks, and the response time. Overall, participants expressed enthusiasm and satisfaction regarding the model. Further refinement is needed to improve the model’s accuracy and diversity of content related to caregiving tasks, level of engagement, recommended videos, and the response time. Our prototype has potential to provide greatly needed personalized support for family caregivers.

